# An open-label, phase 1 study evaluating safety, tolerability, and pharmacokinetics of linifanib (ABT-869) in Japanese patients with solid tumors

**DOI:** 10.1007/s00280-012-1846-6

**Published:** 2012-03-02

**Authors:** Hajime Asahina, Yosuke Tamura, Hiroshi Nokihara, Noboru Yamamoto, Yoshitaka Seki, Takashi Shibata, Yasushi Goto, Maki Tanioka, Yasuhide Yamada, Andrew Coates, Yi-Lin Chiu, Xiaohui Li, Rajendra Pradhan, Peter J. Ansell, Evelyn M. McKeegan, Mark D. McKee, Dawn M. Carlson, Tomohide Tamura

**Affiliations:** 1 Division of Thoracic Oncology, National Cancer Center Hospital (NCCH), 5-1-1 Tsukiji, Chuo-ku, Tokyo, 104-0045 Japan; 2 Abbott Oncology, Abbott Laboratories, Abbott Park, IL USA; 3 Division of Gastrointestinal Oncology, National Cancer Center Hospital (NCCH), 5-1-1 Tsukiji, Chuo-ku, Tokyo, 104-0045 Japan

**Keywords:** Angiogenesis, Linifanib (ABT-869), PDGFR, Solid tumors, VEGFR, Japanese

## Abstract

**Purpose:**

This phase 1 study assessed the safety, tolerability, pharmacokinetics, and preliminary antitumor activity of linifanib in Japanese patients with advanced solid tumors.

**Methods:**

Patients were assigned to one of four sequential cohorts (0.05, 0.10, 0.20, or 0.25 mg/kg) of oral, once-daily linifanib on a 21-day cycle. Adverse events (AEs) were assessed per common terminology criteria for adverse events v3.0; tumor responses were assessed by response evaluation criteria in solid tumors.

**Results:**

Eighteen patients were enrolled. Eleven (61%) received ≥3 prior therapies. Dose-limiting toxicities were Grade 3 ALT increase (0.10 mg/kg linifanib) and Grade 1 T-wave inversion (0.25 mg/kg linifanib) requiring dose interruption for >7 days and discontinuation on day 29. The most common linifanib-related AE was hypertension. Other significant treatment-related AEs included proteinuria, fatigue, and palmar-plantar erythrodysaesthesia. Linifanib pharmacokinetics were dose-proportional across 0.10–0.25 mg/kg. Two patients (11.1%) had confirmed partial responses, 12 had a best response of stable disease (11 had stable disease for ≥12 weeks), and four patients were not evaluable due to incomplete data. Four patients (lung cancer, breast cancer, thymic cancer, sarcoma) have continued linifanib for ≥48 weeks (range, 48–96+ weeks).

**Conclusion:**

Linifanib was well tolerated with promising preliminary clinical activity in Japanese patients. Later-phase global studies examining linifanib efficacy will include Japanese patients.

## Introduction

Angiogenesis is a complex process of vascular network formation essential for growth and metastasis of both normal and tumor cells, supported by vascular endothelial growth factors (VEGF) and platelet-derived growth factors (PDGF) binding to the receptor tyrosine kinases (RTKs) VEGFR and PDGFR [1–4]. Excessive production of VEGF, PDGF, and placental growth factor (PlGF) by solid tumor cells can result in excessive angiogenesis [5], and dysregulation of growth-factor/RTK interactions on tumors and tumor vasculature can result in increased tumor growth and metastasis [4]. Consequently, the inhibition of VEGF, PDGF, and their RTKs is a potential target for cancer therapy [6, 7].

Small-molecule RTK inhibitors constitute the largest category of antiangiogenic anticancer drugs. Three RTK inhibitors, sorafenib, sunitinib, and pazopanib, target multiple receptors including VEGFR and PDGFR, and are approved for treatment in various solid tumor types. Other multiple RTK inhibitors in development for treatment of solid tumors include axitinib, motesanib, vandetanib, cediranib, brivanib, and SU14813. Combined inhibition of VEGFR and PDGFR is hypothesized to have a greater antitumor effect than inhibition of individual receptors [8]. Multiple-targeted RTK inhibitors, however, lack target specificity, which can result in unexpected toxicity, including fatigue, rash, myalgia, and hand-foot syndrome [5].

Linifanib (ABT-869) is a novel, potent inhibitor with selectivity for the VEGFR and PDGFR family of receptor tyrosine kinases. It has specific inhibitory activity against VEGFR-1, VEGFR-2, PDGFRβ, colony-stimulating factor 1 receptor, and fms-related tyrosine kinase 3, with minimal activity against unrelated tyrosine and serine/threonine kinases [9–11]. In preclinical studies with multiple human tumor xenograft models, linifanib demonstrated potent antiangiogenic and antitumor effects [9–13]. In a phase 1 study, single-agent linifanib demonstrated safety and activity in Asian patients with refractory solid malignancies [14]. Linifanib has also shown antitumor activity in phase 2 studies in patients with non-small cell lung cancer [15], hepatocellular carcinoma, or renal cell carcinoma (preliminary results) [16, 17].

This phase 1 study evaluated the pharmacokinetics, safety, and tolerability of linifanib in Japanese patients with solid tumors, at doses similar to those in the phase 1 study in Asian patients [14], and conducted a preliminary assessment of antitumor activity.

## Patients and methods

### Patients

Eligible patients were aged 20–75 years, with a histologically or cytologically confirmed solid tumor refractory to standard therapies or for which a standard effective therapy did not exist, Eastern Cooperative Oncology Group Performance Status (ECOG PS) 0–2, and adequate renal, hepatic, and bone marrow function (absolute neutrophil count ≥1,000/μL, platelets ≥100,000/μL, and hemoglobin ≥9.0 g/dL). Exclusion criteria included body weight ≤41 kg (0.05 and 0.10 mg/kg cohort) or ≥63 kg (0.05 mg/kg cohort), central nervous system metastasis, proteinuria greater than Grade 1 per the National Cancer Institute Common Terminology Criteria for Adverse Events version 3.0 (CTCAE v3) [18], hypertension (systolic/diastolic blood pressure >150/>95 mmHg), left ventricular ejection fraction <50%, and serum positivity for human immunodeficiency virus, or hepatitis B or C virus.

### Study design and treatment

This phase 1, open-label, dose-escalating study [19] was approved by the institutional review boards and ethics committees at the National Cancer Center Hospital (NCCH), and conducted in accordance with the Good Clinical Practice guidelines and the Declaration of Helsinki. All patients gave written informed consent before study-related procedures.

The primary study objective was to evaluate the safety, tolerability, and pharmacokinetics of linifanib in Japanese patients with solid tumors. The secondary objective was to obtain a preliminary assessment of antitumor activity. An exploratory analysis was conducted to identify potential biomarkers that could predict linifanib activity or serve as surrogates for clinic endpoints in future linifanib studies.

A standard 3 + 3 design determined the dose level assignment. Patients were assigned to one of four sequential dose cohorts of once-a-day dose regimen of oral linifanib: 0.05, 0.10, 0.20, or 0.25 mg/kg, administered in the morning. The 0.25 mg/kg dose was the highest dose planned in order to establish a uniform global phase 2 dose, since a prior phase 1 study in non-Japanese resulted in a recommended phase 2 dose of 0.25 mg/kg [14]. Dose-limiting toxicity (DLT) was defined as Grade 4 neutropenia lasting >7 days, Grade 4 thrombocytopenia or decreased hemoglobin, Grade 3 or greater thrombocytopenia (if blood transfusion was required), febrile neutropenia, non-hematological toxicity except for manageable nausea, vomiting, anorexia, diarrhea, constipation or electrolyte abnormality, or a toxicity that required suspension of study drug for >7 days.

Patients self-administered linifanib once daily, on a 21-day cycle after fasting, and treatment continued until disease progression or intolerable toxicity. Patients were discontinued from study participation if they exhibited disease progression, had linifanib-related toxicities requiring >2 weeks of dose interruption, or required alternate antineoplastic therapy. The initial oral dose, 2.5–25.0 mg in increments of 2.5 mg, was determined by the patients’ weights. At each dose reduction, the linifanib dose was generally decreased by 2.5 mg. The dose was reduced by 5.0 mg for patients ≥86 kg in the 0.10 mg/kg cohort, for patients in the 0.20 mg/kg cohort who were ≥81 kg at the first reduction and 61–80 and ≥96 kg at the 2nd reduction, and for patients in the 0.25 mg/kg cohort who were ≥66 kg at the first reduction and ≥86 kg at the second reduction. Patients were discontinued if they required dose reduction, specified by cohort: Any reduction (0.05 mg/kg cohort); >1 reduction (0.10 mg/kg cohort, and patients ≤31 kg in the 0.20 mg/kg cohort with an initial 5 mg dose); >2 reductions (0.25 mg/kg cohort, and patients ≥32 kg in the 0.20 mg/kg cohort with an initial ≥7.5 mg dose).

### Tumor response and safety

Baseline evaluations included physical examination, body weight, vital signs, 12-lead electrocardiogram, ECOG PS assessment, pregnancy test, laboratory tests, and multiple-gated acquisition scan/echocardiogram. Tumor response and/or disease progression was assessed by computerized tomography (CT) scan or magnetic resonance imaging (MRI) per RECIST [20] at screening and on Day (D) 1 of every second cycle prior to the subsequent treatment period, until tumor progression or until final visit. Complete response and partial response (PR) were defined according to RECIST [20]; objective response rate (ORR) was defined as the proportion of patients with best response of PR or CR among the study population. Safety assessments included laboratory test results and adverse events (AEs), which were graded according to CTCAE v3 [18] and coded by medical dictionary of regulatory activities (MedDRA) 1.0.

### Pharmacokinetic and pharmacodynamic assessments

Pharmacokinetic sampling occurred pre-dose and 0.5, 1, 2, 3, 4, 6, 8, and 24 h after single-dose linifanib on Cycle (C) 1D1, and pre-dose and 0.5, 1, 2, 3, 4, 6, and 8 h after multiple once-daily doses on C1D15. Urine was collected for 24-h after the C1D15 dose. Linifanib and its metabolite concentrations in plasma and urine were determined using a validated method based on triple quadruple tandem mass spectrometry with a lower limit of quantification of 1.0 ng/mL.

Pharmacokinetic parameter (defined in Table 3) concentrations were determined by non-compartmental analysis using WinNonlin Professional v.5.2 (Pharsight Corp., Cary, NC). Dose proportionality was evaluated by linear regression analysis for dose-normalized (DN) maximum observed plasma concentration (*C*_max_) and DN area under the plasma concentration–time curve 0–24 h (AUC_24_) on C1D1, and DN *C*_max_ and DN AUC_24_ on C1D15 across doses 0.05, 0.1, 0.2, and 0.25 mg/kg. Additional samples were collected at C3D1 (pre-dose) and every second cycle until study completion or until C15D1. Concentrations for samples at C3D1 and subsequent samples, and data from C1D1 to C1D15 were included in the nonlinear mixed effects models to explore covariates such as age, body weight, and gender (data not shown). Following single-dose linifanib at 0.25 mg/kg, a post hoc analysis compared the pharmacokinetics between the Japanese patients in the current study and non-Japanese patients in two phase 1 studies: Caucasian patients receiving 0.25 mg linifanib (Abbott, unpublished) and the non-Japanese segment of Asian patients receiving 0.10–0.30 mg/kg linifanib [14].

Plasma for biomarker analysis was collected before linifanib administration on C1D1, C1D15, C2D1, and at the final visit. Concentration of PlGF was determined using Abbott Architect^®^ kits. The relationship of PlGF levels to outcomes was assessed post hoc. To assess the relationship between PlGF induction and toxicity, patients were grouped into those requiring and not requiring dose interruption during the first 30 days of therapy. Median PlGF increase from baseline to C1D15 was compared as a function of toxicity group. To assess the relationship between PlGF induction and efficacy, patients were segregated into those with progressive disease (PD; *N* = 6) or stable disease (SD; *N* = 10) at C6, and PlGF increase from baseline to C1D15 was compared.

### Statistical analysis

Continuous variables from clinical data were summarized by the number of observations, mean, standard deviation, median, minimum, and maximum. Discrete variables were summarized by frequency and proportion. Statistical significance for clinical and pharmacodynamic analyses was determined by a 2-sided *P* value <0.05.

## Results

### Patient characteristics

From September 2008 to September 2009, 18 patients with various solid tumor types were enrolled at the NCCH in Japan. Initial linifanib doses in each patient were 0.05 mg/kg (*n* = 3), 0.10 mg/kg (*n* = 6), 0.20 mg/kg (*n* = 3), and 0.25 mg/kg (*n* = 6). Patient baseline and disease characteristics were well balanced across the dose groups (Table 1). The majority were women, had ECOG PS of 0, and had received three or more prior systemic therapies (Table 1). Median (range) treatment duration was 147 days (7–672+). Median (range) dose intensity, defined as the percent of full-dose daily linifanib received from C1D1 to treatment discontinuation, was 91% (33–100).

**Table 1:** Patient and disease characteristics

Baseline characteristics	All patients *N* = 18	Linifanib dose, mg/kg			
		0.05 *n* = 3	0.10 *n* = 6	0.20 *n* = 3	0.25 *n* = 6
Median age (range), years	52 (38–69)	62 (47–64)	50 (38–62)	61 (42–62)	53 (39–69)
Gender, *n* (%)					
Male	6 (33.3)	0	2 (33.3)	0	4 (66.7)
Female	12 (66.7)	3 (100)	4 (66.7)	3 (100)	2 (33.3)
Median body weight, kg	56.5	47.3	56.5	58.1	64.0
ECOG PS^a^, *n* (%)					
0	10 (55.6)	1 (33.3)	4 (66.7)	3 (100)	2 (33.3)
1	8 (44.4)	2 (66.7)	2 (33.3)	0	4 (66.7)
Type of primary cancer, *n* (%)					
Lung	8 (44.4)	1 (33.3)	3 (50.0)	0	4 (66.7)
Sarcoma	5 (27.8)	2 (66.7)	2 (33.3)	0	1 (16.7)
Breast	3 (16.7)	0	0	3 (100)	0
Others^b^	2 (11.1)	0	1 (16.7)	0	1 (16.7)
Prior systemic therapies, *n* (%)					
0–2	7 (38.9)	1 (33.3)	3 (50.0)	0	3 (50.0)
≥3	11 (61.1)	2 (66.7)	3 (50.0)	3 (100)	3 (50.0)
Smoker, *n* (%)					
Current or ever	6 (33.3)	0	3 (50.0)	0	3 (50.0)

*ECOG PS* Eastern Cooperative Oncology Group performance status

^a^No patients had ECOG PS ≥2

^b^Other types of primary cancers included thymic cancer (*n* = 1, 0.10 mg/kg) and colon cancer (*n* = 1, 0.25 mg/kg)

### Safety and tolerability

The most common linifanib-related AEs were hypertension, increased aspartate aminotransferase (AST), rash, neutropenia, and increased blood triglycerides (Table 2). There were no Grade 3 linifanib-related AEs at the 0.05 mg/kg dose, three at 0.10 mg/kg, two at 0.20 mg/kg, and four at 0.25 mg/kg. Grade 3 linifanib-related AEs included proteinuria (*n* = 4), neutropenia (*n* = 2), increased alanine aminotransferase (ALT) (*n* = 2), diarrhea, increased blood magnesium, decreased lymphocyte count, and hypertension. There were no Grade 4 or 5 AEs. Two DLTs were reported. One patient (0.10 mg/kg cohort) had a Grade 3 ALT increase, and one (0.25 mg/kg cohort) had a Grade 1 T-wave inversion requiring dose interruption for >7 days and discontinuation on D29.

**Table 2:** Linifanib-related adverse events by dose and grade level

	Linifanib doses, mg/kg												All patients *N* = 18 (%)
	0.05 *n* = 3			0.10 *n* = 6			0.20 *n* = 3			0.25 *n* = 6			
	G1	G2	G3	G1	G2	G3	G1	G2	G3	G1	G2	G3	Any grade
Linifanib-related AEs in ≥40% of patients													
Hypertension	1	2			5			2	1		6		17 (94)
Rash	1			3			1			6			11 (61)
Proteinuria				1	2	1			1	1	2	2	10 (56)
Weight decreased	2			2	1		1	2		1	1		10 (56)
Fatigue				1	1		1	1		4	1		9 (50)
Palmar-plantar erythrodysaesthesia					3			3			3		9 (50)
Diarrhea	1			3			1		1		2		8 (44)
Hematological													
Neutropenia	2			1		1		2		2	2	1	11 (61)
Leukopenia				2	1		2			3	2		10 (56)
Thrombocytopenia				1	1			2		2	2		8 (44)
Blood Chemistry													
AST increased	1			4			2			6			13 (72)
Blood TG increased	1			3	1		3			2	1		11 (61)
ALT increased	1			2		1*	2			3		1	10 (56)
Blood cholesterol increased	2			3			2	1		2			10 (56)
Blood urine present	1			2			2			4			9 (50)
Blood TSH increased				3			3			1	1		8 (44)
GGT increased				3	1		1			3			8 (44)
Blood ALKP increased	1	1		1			1			4			8 (44)
Other AEs of Interest													
Anorexia	1			2			1	1			2		7 (39)
T-wave abnormality							1			1^a^			2 (11)

No grade 4 or 5 toxicities were observed or reported

*AST* aspartate aminotransferase, *TG* triglycerides, *ALT* alanine aminotransferase, *TSH* thyroid stimulating hormone, *GGT* gamma glutamyltransferase, *ALKP* alkaline phosphatase

^a^Dose-limiting toxicity

Adverse events leading to dose reductions were palmar-plantar erythrodysaesthesia (*n* = 2), abdominal pain, abdominal pain upper, diarrhea, gastritis, increased ALT, and decreased platelet count. Adverse events leading to dose interruptions in two or more patients were palmar-plantar erythrodysaesthesia (*n* = 4), decreased platelet count (*n* = 3), abdominal pain upper (*n* = 3), diarrhea (*n* = 2), fatigue (*n* = 2), increased ALT (*n* = 2), and proteinuria (*n* = 2). There were no dose reductions or interruptions for hypertension, neutropenia, or leucopenia. Of 16 patients who discontinued the study, 12 discontinued due to PD, one due to PD and AE, two due to AEs, and one due to an AE and withdrawal of consent.

### Pharmacokinetics

Pharmacokinetic data were available for 18 and 16 patients on C1D1 and C1D15, respectively. Table 3 shows the pharmacokinetic parameters following linifanib single dose or multiple daily doses. Linifanib was rapidly absorbed, with average *T*_max_ approximately 2 h across all dose levels. Patients receiving the lowest dose had slightly higher DN exposures over 24-h post-administration (DN AUC_24_). Comparison of DN pharmacokinetic data across the 0.10, 0.20, and 0.25 mg/kg cohorts revealed no significant trend with dose level in peak serum concentration (DN *C*_max_) or DN AUC_24_ on C1D1 or C1D15 (*P* > 0.05). The DN AUC_24_ on C1D15 was approximately 1.5-fold of the DN AUC_24_ on C1D1 for each dose level (accumulation ratio approximately 1.5). The effective half-life of linifanib after repeated daily dosing associated with this value is 15 h (Table 3). Of 13 patients with available urine data, <15% of the dose was recovered as unchanged drug and metabolite across doses. Post hoc analysis showed that the pharmacokinetics for the Japanese patients following a single dose in the current study were similar to those of non-Japanese subjects in historical linifanib studies (Table 4).

**Table 3:** Mean ± SD linifanib pharmacokinetic parameters after single (study day 1) and multiple (study day 15) doses of linifanib

Pharmacokinetic parameters, units	Linifanib (mean ± SD)			
	0.05 mg/kg	0.10 mg/kg	0.20 mg/kg	0.25 mg/kg
	Single dose			
*N*	3	6	3	6
*T*_max_ (h)	1.65 ± 0.56	1.67 ± 0.52	1.67 ± 0.57	2.33 ± 1.03
*C*_max_ (μg/mL)	0.09 ± 0.018	0.152 ± 0.036	0.305 ± 0.070	0.305 ± 0.068
DN *C*_max_ (μg/mL/mg)	0.036 ± 0.007	0.026 ± 0.004	0.028 ± 0.007	0.019 ± 0.004
AUC_24_ (μg h/mL)	1.23 ± 0.21	1.91 ± 0.38	3.60 ± 0.43	3.78 ± 0.48
DN AUC_24_ (μg h/mL/mg)	0.49 ± 0.08	0.33 ± 0.04	0.33 ± 0.01	0.25 ± 0.02

	Multiple dose			
*N*	3	5	3	5
*T*_max_ (h)	2.34 ± 0.57	2.20 ± 0.45	2.33 ± 0.58	2.20 ± 0.45
*C*_max_ (μg/mL)	0.128 ± 0.017	0.186 ± 0.064	0.390 ± 0.041	0.418 ± 0.055
DN *C*_max_ (μg/mL/mg)	0.051 ± 0.007	0.034 ± 0.008	0.036 ± 0.003	0.028 ± 0.002
AUC_24_^a^ (μg h/mL)	1.92 ± 0.45	2.82 ± 1.03	5.63 ± 0.92	5.46 ± 1.16
DN AUC_24_ (μg h/mL/mg)	0.77 ± 0.18	0.50 ± 0.11	0.52 ± 0.02	0.37 ± 0.06
*R* ^b^	1.58 ± 0.34	1.46 ± 0.27	1.56 ± 0.08	1.46 ± 0.23

*SD* standard deviation, *T*_max_ time to *C*_max_, *h* hour, *DN* dose-normalized, *C*_max_ maximum observed plasma concentration, AUC_24_ area under the concentration time curve 0–24 h

^a^AUC_24_ on C1D15 was calculated assuming the pre-dose concentration is equal to the concentration at 24 h post-dose because no 24-h pharmacokinetic sample was drawn following the C1D15 dose

^b^Accumulation ratio calculated as DN AUC_24_ between D15 and D1

**Table 4:** Pharmacokinetic comparison between Japanese and non-Japanese patients with solid tumors after single doses of linifanib

Pharmacokinetic parameter	Linifanib (mean ± SD)		
	Japanese	Caucasian^a^	Asian^b^
	0.25 mg/kg	0.25 mg/kg	0.10–0.30 mg/kg
*N*	6	13	31
*T*_max_ (h)	2.33 ± 1.03	1.77 ± 0.44	2.94 ± 1.27
DN *C*_max_ (μg/mL/mg)	0.019 ± 0.004	0.018 ± 0.005	0.020 ± 0.008
DN AUC_24_ (μg h/mL/mg)	0.25 ± 0.02	0.21 ± 0.04	0.25 ± 0.10

*SD* standard deviation, *T*_max_ time to *C*_max_, *h* hour, *DN* dose-normalized, *C*_max_ maximum observed plasma concentration, AUC_24_ area under the concentration time curve 0–24 h

^a^Data are from a linifanib phase 1 study [33]

^b^Data for these non-Japanese Asian patients (Chinese, Malay, Indian, Arab) were calculated from a linifanib phase 1 study [14]

### Efficacy

**Fig. 1:**
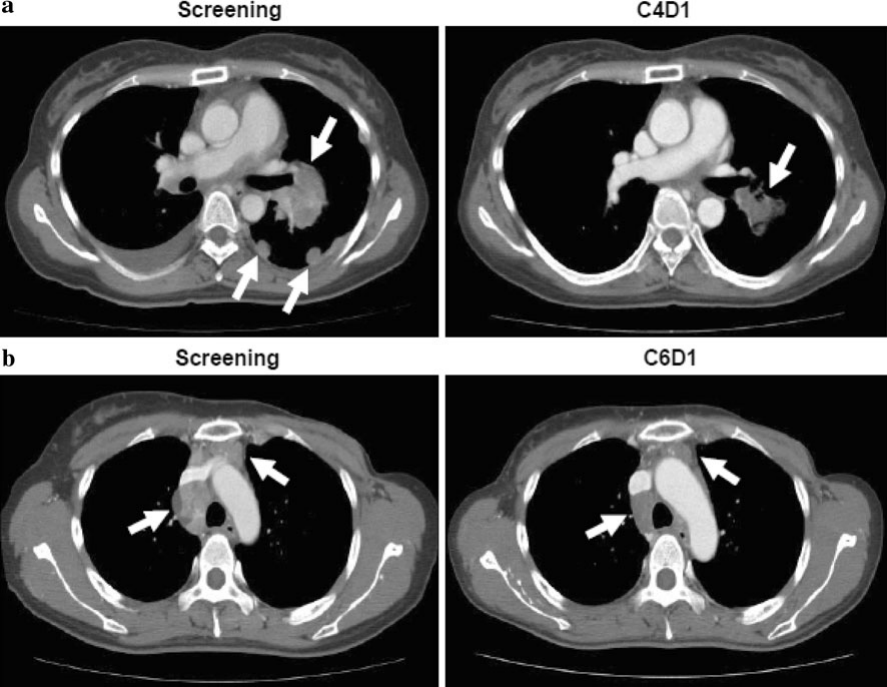
CT images for two patients with confirmed partial responses following linifanib treatment. **a** A 39-year-old female patient with lung cancer who received prior chemotherapy had lesions in the lung, pleura, and lymph nodes. This patient had a confirmed PR in C4, following linifanib treatment at 0.25 mg/kg. *Arrows* indicate tumor location at screening and at C5D1. **b** A 42-year-old female patient with breast cancer who received prior chemotherapy had target lesions in the mediastinal lymph nodes. This patient had a confirmed PR in C2, following linifanib treatment at 0.20 mg/kg. *Arrows* indicate tumor location at screening and at C6D21. Abbreviations: *CT* computerized tomography; *PR* partial response; *C* cycle; *D* day

**Fig. 2:**
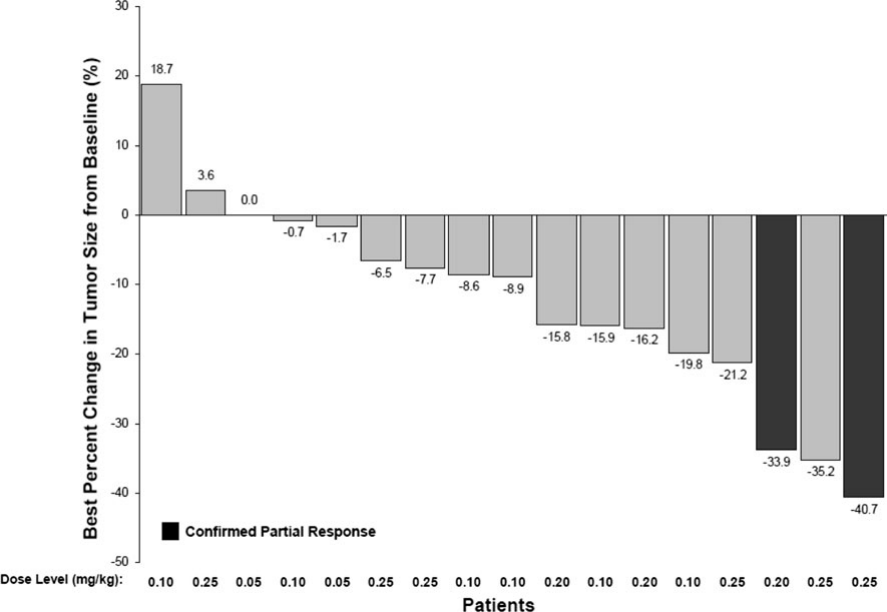
Best percentage change from baseline in tumor size in patients treated with linifanib. Data for 17 of 18 patients are shown. Of the 18 patients in this study, one patient had no measurable lesions at baseline. This patient was not evaluable due to incomplete data

Two patients had confirmed PRs. One of these had breast cancer and received treatment in the 0.20 mg/kg cohort for 147 days. The other had lung cancer and received treatment in the 0.25 mg/kg cohort for 131 days. Figure 1 shows representative CT scans for these two patients. The ORR was 2 of 18 patients, 11.1%. Twelve patients had SD. Of these, 11 had SD for ≥12 weeks, including patients with lung cancer, breast cancer, sarcoma, thymic cancer, and colon cancer. Tumor response was not evaluable in four patients; one had tumors that were not measurable at baseline, one had tumors that were not measurable after treatment, and two had early discontinuation due to AEs or clinical deterioration. Median (range) progression-free survival (PFS) was 5.7 months (2.8–9.8). Median (range) duration of response was 3.2 months (2.8–3.5). The best tumor response at imaging assessments for each patient is illustrated in Fig. 2. A reduction in summed tumor dimensions of ≥5% was seen in 12 of the 18 patients on study and in cohorts 0.10, 0.20, and 0.25 mg/kg.

Four patients continued linifanib with clinical benefit for ≥48 weeks (range, 48–96+ weeks). These patients had sarcoma, breast cancer, lung cancer, and thymic cancer. All had a best tumor response of SD. A post hoc analysis showed that pharmacokinetic parameter values and PlGF levels for these four patients were not notably different from the levels for the other patients in the study (data not shown).

### Pharmacodynamics

**Fig. 3:**
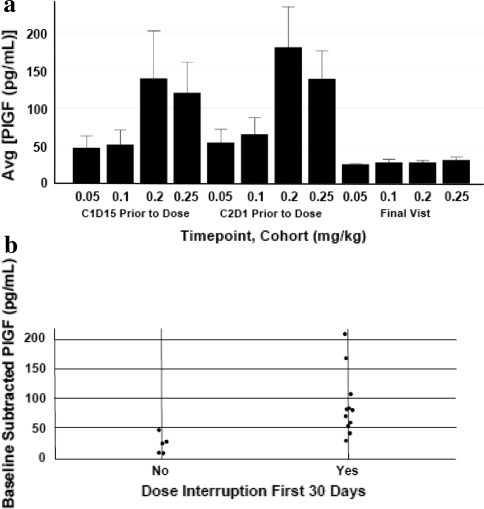
Baseline subtracted day 15 placental growth factor (PlGF). **a** Average PlGF increase from baseline by dose cohort. **b** PlGF increase from baseline to C1D15 in patients who required a dose interruption during the first 30 days of therapy compared with patients who did not. *Avg* average; *C* cycle; *D* day

Induction of PlGF was observed on C1D15 and C2D1 upon treatment with linifanib at a dose-dependent fashion. Concentration of PlGF returned to near baseline levels at the final visit when patients were no longer on therapy, indicating PlGF increase is reversible (Fig. 3a).

To explore relationships between PlGF induction and toxicity, PlGF changes from baseline to C1D15 were compared in patients who required a dose interruption during the first 30 days of therapy and those who did not. The median (±SD) increase from baseline to C1D15 PlGF was 22.8 pg/mL (±16.2) for the five patients who did not need a dose interruption and was 79.9 (±55.0) for the 11 patients who did (Fig. 3b). Taken together, these data indicate PlGF induction is dose-dependent. To examine a relationship between PlGF induction and efficacy, patients were segregated into those with PD (*n* = 6) or SD (*n* = 10) at C6. No statistically significant difference in PlGF change from baseline to C1D15 as a function of response classification (*p* = 0.7) was observed.

## Discussion

The results of this phase 1 study showed that linifanib had a favorable safety profile in this Japanese population. Patients had minimal DLTs (two) and no Grade 4 AEs. Toxicities were mild to moderate and were manageable. The most frequently observed toxicity was hypertension, which occurred in 17 of the 18 patients across all dose groups. All events of hypertension were Grade 1 or 2, except for one instance of Grade 3 (0.20 mg/kg dose). Although other phase 1 TKI studies in Japanese patient populations have reported Grade 3 hypertension as an adverse event [21–23], a direct comparison with the current study is difficult due to the small number of patients in the other studies, and differences to the current study in their dose escalation designs. In the phase 1 linifanib study in non-Japanese Asian patients [14], Grade 3 hypertension was observed in 8% of patients at the recommended phase 2 dose, and in other, mixed-population, TKI phase 1 studies, including cediranib [24], motesanib [25], and brivanib [26], Grade 3 hypertension was observed in 14–20% of patients at the phase 2 recommended dose levels. The most common linifanib-related AEs in the present study (hypertension, rash, neutropenia, proteinuria, weight decreased, leukopenia, fatigue, palmar-plantar erythrodysaesthesia) as well as linifanib-related Grade 3 AEs (proteinuria, diarrhea, neutropenia, increased ALT, and increased blood magnesium) were comparable to the most common drug-related AEs in other phase 1, dose-escalating studies in multi-targeted TKIs [14, 21–32]. The phase 1 linifanib trial in Asian patients showed that linifanib-related toxicities increased in frequency and intensity with increasing doses, hypertension was dose-dependent, patients responded to antihypertensive therapy, and proteinuria and skin blisters resolved after reduction or stopping linifanib dosing. In the present study, dose interruption or reduction was seen for Grade 2 palmar-plantar erythrodysaesthesia and Grade 3 proteinuria; however, a relationship between the linifanib dose level and AE incidence could not be established due to the small number of patients in each dose group.

The 18 Japanese patients in this study received oral linifanib daily at escalating doses of 0.05, 0.10, 0.20, and 0.25 mg/kg. Linifanib was rapidly absorbed with an average *T*_max_ of approximately 2 h across all dose levels. After 15 days of repeated daily dosing, linifanib accumulated 1.5-fold and the effective half-life was approximately 15 h. The urinary excretion of linifanib was a minor pathway following oral administration. Similar *T*_max_ and half-life were seen in non-Japanese linifanib phase 1 studies [14, 33]. Daily doses ≥0.1 mg/kg used in the current study achieved the efficacious plasma exposures at steady state (≥2.7 μg h/mL) predicted based on a preclinical murine HT1080 fibrosarcoma model [10]. The pharmacokinetics following single-dose administration at 0.25 mg/kg from this Japanese study are similar to those from the non-Japanese phase 1 studies [14, 33]. Linifanib pharmacokinetics were dose-proportional over the 0.10–0.25 mg/kg single and once-daily dose range, also reported in the linifanib phase 1 dose-escalating trial in non-Japanese patients [14].

Circulating levels of PlGF, which increase with VEGFR inhibition, have the potential to act as a pharmacodynamic biomarker [34]. In a previous phase 1 linifanib study, PlGF increased dose-dependently [35]. This study confirmed the dose-dependent increase in PlGF following linifanib therapy and demonstrated that larger increases in PlGF concentrations were observed in patients requiring a dose reduction. In a post hoc analysis of the four patients on this study ≥48 weeks, PlGF was not notably different compared with the other study patients.

Conclusions regarding efficacy in phase 1 studies are necessarily limited. Although tumor evaluation was not the primary objective of this study, linifanib demonstrated encouraging preliminary antitumor activity across a range of tumor types (lung cancer, breast cancer, colon cancer, and others). Tumor reduction >5% by RECIST was observed in the majority of patients (12/18, 67%), and PRs were observed in two patients at the 0.20 mg/kg and 0.25 mg/kg dose levels. The four patients participating in the present study for ≥48 weeks have received 0.05 mg/kg, 0.10 mg/kg, or 0.20 mg/kg linifanib; three had a decrease in tumor size from baseline, and none had Grade 3 or 4 linifanib-related AEs. Substantial conclusions about clinical efficacy cannot be made due to the small size of the population. Preliminary antitumor activity was also demonstrated in a phase 1 trial of linifanib for solid tumors in Asian patients [14] and in three phase 2 trials of linifanib for solid tumors [15, 16, 36]. Similar, preliminary, antitumor efficacy has been seen in phase 1 studies of other TKIs [21–28, 30, 32].

In summary, linifanib was well tolerated in Japanese patients with solid tumors at the dose range 0.05–0.25 mg/kg. Linifanib pharmacokinetics were dose-proportional at the 0.10–0.25 mg/kg dose range following single and multiple once-daily oral administration. The pharmacokinetics of Japanese patients following single-dose administration at 0.25 mg/kg are similar to those seen in non-Japanese patients. Dose-dependent increases in PlGF were observed, but did not demonstrate a clear association with patient response to linifanib.
